# A case of massive hemoptysis caused by immunoglobulin G4-related respiratory disease in adults: case report and review of literature

**DOI:** 10.3389/fimmu.2024.1432508

**Published:** 2024-12-20

**Authors:** Chun-xia Mei, Guo-long Yue, Xia Feng, Hai-qiao Wu, Jiong Li

**Affiliations:** Department of Respiratory, Chongqing Hospital of Traditional Chinese Medicine, Chongqing, China

**Keywords:** immunoglobulin G4-related disease, immunoglobulin G4-related respiratory disease, hemoptysis, treatment, prognosis

## Abstract

Immunoglobulin G4-related disease (IgG4-RD) is an immune-mediated chronic fibro-inflammatory condition, that can involve multiple systems. Immunoglobulin G4-related respiratory disease (IgG4-RRD) is relatively rare, with non-specific clinical symptoms. Hemoptysis is a rare clinical symptom of IgG4-RRD, and cases of massive hemoptysis in adults have not been reported. We present here a rare case of massive hemoptysis caused by IgG4-RRD in adults and review relevant literature. An 84-year-old female presented with recurrent cough and blood-streaked sputum, progressing to massive hemoptysis. Her chest CT showed patchy lesions in the lungs, initially misdiagnosed as a tumor. Ultimately, a biopsy confirmed the diagnosis of IgG4-RRD. The patient was treated with prednisone combined with leflunomide, which controlled her condition and maintained remission. However, after 13 months without hemoptysis, she experienced intermittent hemoptysis followed by a massive episode. Increasing the prednisone dose and continuing leflunomide treatment controlled the condition once again, with no recurrence in the subsequent year of follow-up. In patients with IgG4-RRD, particularly those with hemoptysis, it is essential to remain vigilant for massive hemoptysis. Similarly, in patients with lung patch lesions and no evidence of a tumor on biopsy, IgG4-RRD should not be overlooked. Early diagnosis and timely treatment can improve the patient’s clinical prognosis.

## 1 Introduction

ImmunoglobulinG4-related disease (IgG4-RD) is a recently recognized immune-mediated chronic fibro-inflammatory condition (1). It is marked by swelling or mass formation in various organs with infiltration of IgG4-positive plasma cells. The disease can affect virtually any organ either singly or in association (2). Immunoglobulin G4-related respiratory disease (IgG4-RRD) is relatively uncommon compared to other organ involvement in IgG4-RD (3). Isolated lung involvement was estimated to occur in 8% of patients in a Japanese study involving 4,304 individuals (4). A large Chinese cohort study of 448 cases revealed that 23.2% of all IgG4-RD patients were identified as IgG4-RRD (5).

Clinical symptoms of IgG4-RRD are nonspecific, leading to frequent misdiagnosis. The symptoms include cough, sputum production, dyspnea, chest pain, fever, asthenia, weight loss, and hemoptysis (6), with hemoptysis being relatively rare. To date, only one case of massive hemoptysis in pediatric IgG4-RD has been described (10), yet, no cases of massive hemoptysis in adults have been reported before. Here, we report a rare case of an 84-year-old woman diagnosed with IgG4-RRD with massive hemoptysis and provide a comprehensive literature review.

## 2 Case presentation

**Figure 1:**
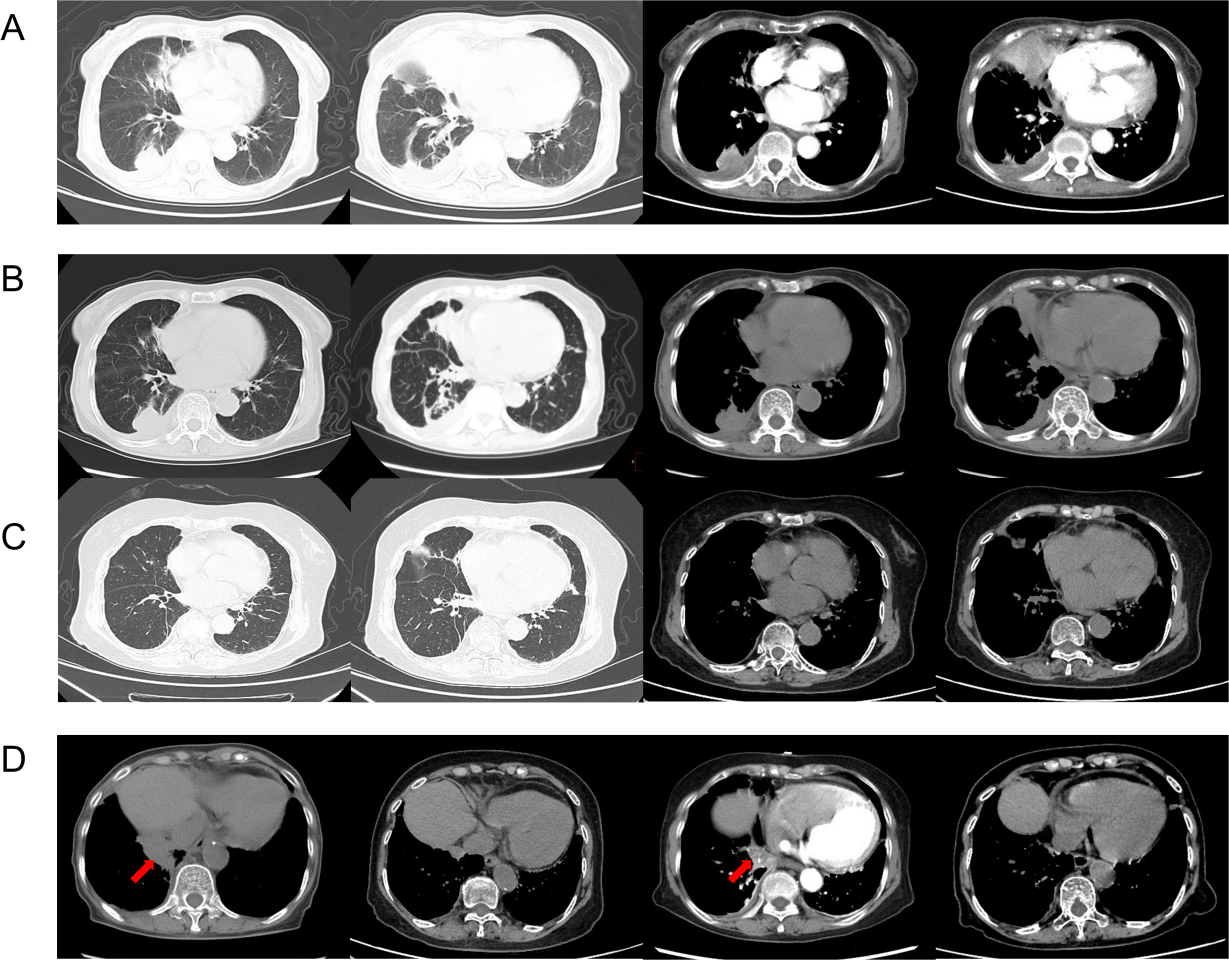
CT chest on admission (2021.3.15) showed patchy density shadows, thickened bronchovascular bundles, pleural effusion, and pleural thickening **(A)**. CT chest of the third admission (2021.5.5) **(B)**. Chest CT scans at 16 months after treatment showed significant absorption of the lung lesions (2022.9.24) **(C)**. The changes in the recurrence sites (arrows) before and after treatment (2021.5.5, 2022.9.24, 2023.1.18, 2023.5.18) **(D)**.

**Figure 2:**
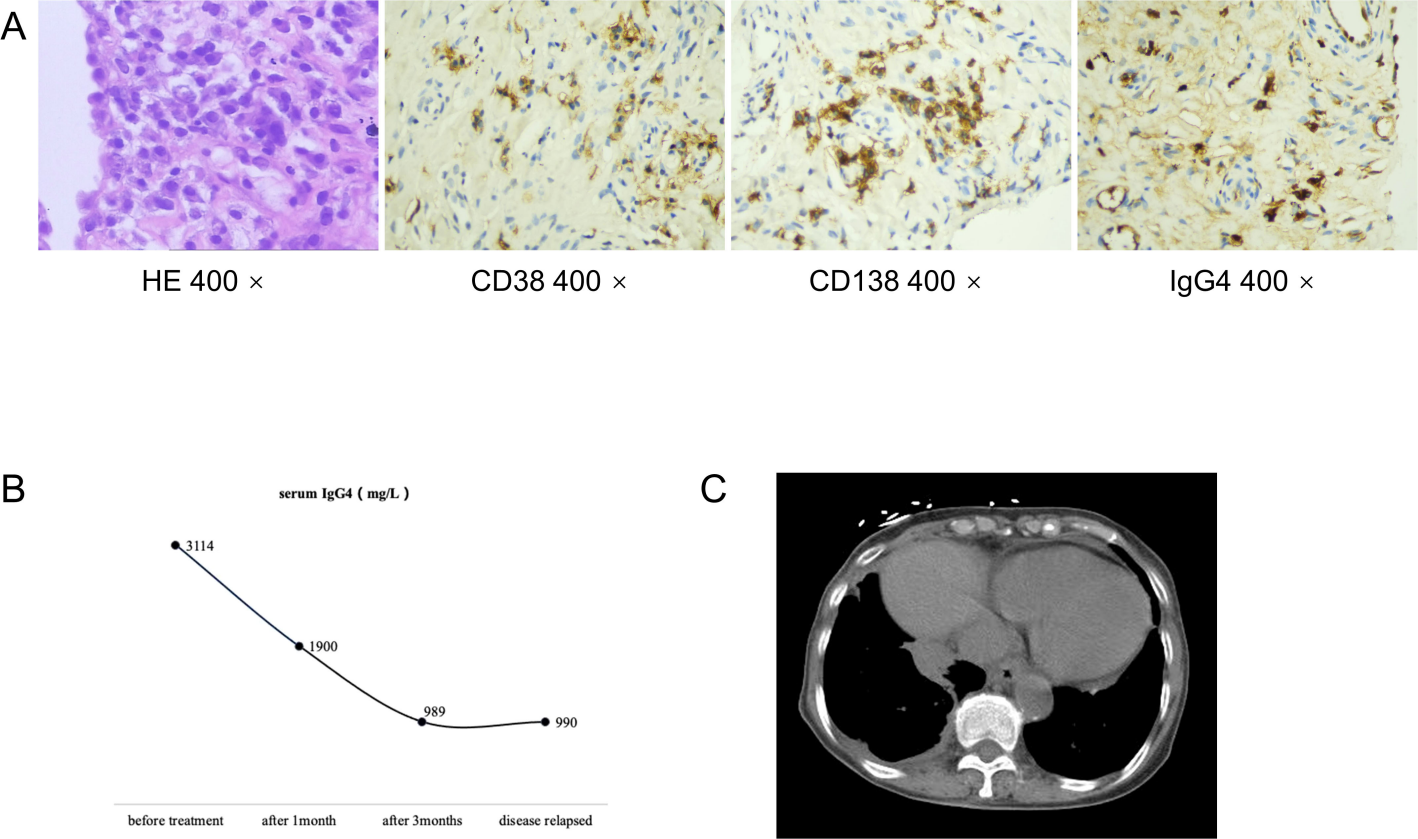
Pathological biopsy of of the right middle lobe showed lymphocyte and plasma cell infiltration, with no tumor cells in the tissue (HE 400x). Immunohistochemical staining revealed infiltration of CD38-positive plasma cells, CD138-positive plasma cells and IgG4-positive plasma cells(CD38 400x, CD138 400x, IgG4 400x) **(A)**. Serum levels of IgG4 during the follow-up **(B)**. CT chest showed paravertebral soft tissue shadows of this patient **(C)**.

An 84-year-old woman, with a history of a thyroid fine-needle aspiration biopsy for left-sided neck swelling one year ago (specific details undisclosed), was admitted for the first time three years ago (March 4, 2021). Her admission was prompted by a six-month history of coughing, with bloody sputum for one month, alongside chest pain, shortness of breath and a single episode of fever. Physical examination on admission revealed a temperature of 36.8°C, heart rate of 107 beats per minute, respiratory rate of 24 breaths/min, blood pressure of 138/70 mmHg, and oxygen saturation of 97%. Decreased tactile vocal fremitus, dullness on percussion, and decreased breath sounds were noted over the right lung, without crackles or wheezes bilaterally. Complete blood count revealed RBC 3.16 × 10^9/L (reference: 3.8-5.1) and Hemoglobin 90 g/L (reference: 115-150). Inflammatory markers showed C-reactive protein (CRP) at 154.24 mg/L (reference: 0-10) and serum amyloid A(SAA) above 320 mg/L (reference: 0-10), while procalcitonin (PCT) was normal. NT-proBNP was 1489.9 pg/mL (reference: 0-250). Tumor markers were negative. Thyroid function showed TSH at 11.881 mIU/L (reference: 0.55-4.78). Liver function tests showed albumin at 28.2 g/L (reference: 40-55) and globulin at 45.1 g/L (reference: 20-40). D-dimer was 2.04 mg/L (reference: 0-1). Coagulation function, renal function, electrolytes, blood lipids, routine stool and urine tests, cardiac enzymes, and cardiac markers were all normal. Immunological tests for HIV, HBsAg, and HCV were negative. Serum (1, 3)-β-D-glucan test (G test) and galactomannan test (GM test), nine respiratory pathogen IgM antibodies, sputum for acid-fast bacilli, and sputum culture were all negative. The ECG was unremarkable. Cardiac ultrasound showed moderate tricuspid regurgitation, mild mitral and aortic regurgitation, increased pulmonary artery pressure (SPAP 46 mmHg), decreased left ventricular diastolic function, and trace pericardial effusion. Chest ultrasound revealed small bilateral pleural effusions. Thyroid ultrasound revealed cystic masses in both lobes of the thyroid (TI-RADS category 2), isoechoic and mixed-echoic nodules in the right thyroid lobe (TI-RADS category 3), hyperechoic lesions in the left lobe (possibly calcifications). Other ultrasound, including abdominal, lower extremity venous, and cervical vascular ultrasounds, showed no abnormalities. The CT chest showed patchy density shadows, thickened bronchovascular bundles, pleural effusion, and pleural thickening (**Figure 1A**). Initial diagnoses upon admission were pneumonia and possible malignant tumor in the right middle lobe. The patient was treated with antibiotics and underwent percutaneous lung biopsy of the right middle lobe. Pathology revealed no tumor cells (**Figure 2A**). The lung tissue showed lymphocyte and plasma cell infiltration, with fibrous tissue proliferation in the alveolar septa. The patient experienced no further hemoptysis and opted for discharge after improvement of the cough. One month after discharge, the patient experienced recurrent hemoptysis, with a total of approximately 500 ml of fresh blood in 24 hours, prompting the second admission (April 14, 2021). Blood tests indicated a further decline in hemoglobin levels: RBC 2.75 × 10^9/L (reference: 3.8-5.1), Hemoglobin 80 g/L (reference: 115-150). CRP was 81.6 mg/L (reference: 0-10), SAA was 262.42 mg/L (reference: 0-10), erythrocyte sedimentation rate (ESR) was 57 mm/h (reference: 0-20), and liver function showed albumin 34.7 g/L (reference: 40-55), prealbumin 68 mg/L (reference: 180-350), and globulin 43 g/L (reference: 20-40). D-dimer was 1.8 mg/L (reference: 0-1). Extractable nuclear antigen (ENA) and anti-neutrophil cytoplasmic antibodies (ANCA) were negative. Other tests showed no significant changes compared to previous results. Imaging was consistent with previous findings. After treatment with antibiotics and hemostatic drugs, the patient had no further hemoptysis and requested discharge. However, eight days after discharge, the patient experienced two episodes of hemoptysis, with approximately 2-3 ml of fresh blood each time, accompanied by fatigue and dizziness, prompting a third admission on May 4, 2021. Laboratory tests revealed a complete blood count within normal limits except for a slightly decreased hemoglobin level 93g/L (reference: 115-150). ESR was 66 mm/h(reference: 0-20), CRP and PCT were normal. Liver function tests were normal except for an elevated globulin level 44.5g/L (reference: 20-40) and decreased albumin/globulin ratio 0.88(reference: 1.2-2.4). Cardiac ultrasound revealed moderate tricuspid regurgitation, elevated pulmonary arterial systolic pressure (SPAP: 36 mmHg), and decreased left ventricular diastolic function (E/F 70%, E/A <0.8). Chest CT findings are depicted in the accompanying image (**Figure 1B**). We conducted a comprehensive immunoglobulin analysis that revealed elevated levels of IgG at 28.49 g/L (reference: 8.6-17.4) and IgG4 at 3114 mg/L (reference: 39.2-864.0). Complement C3 and C4 levels were normal, along with rheumatoid factor, T-cell subpopulations, and antibodies against SSA, SSB, double-stranded DNA, RNP, Sm, Jo-1, Scl-70, and phospholipase A2 receptor. All of these autoimmune antibodies tested negative. Immunohistochemical staining revealed infiltration of CD38-positive plasma cells, CD138-positive plasma cells and IgG4-positive plasma cells. Immunohistochemical staining (×400) showed that IgG4-positive plasma cells were above 10 per high power field (**Figure 2A**).

This patient meets the comprehensive diagnostic criteria for IgG4-RD established in Japan in 2011 (7), as well as the classification criteria formulated by the American College of Rheumatology (ACR) and the European League Against Rheumatism (EULAR) in 2019 (8). After excluding malignancies, connective tissue diseases, systemic vasculitis, chronic infections, allergic diseases, inflammatory myofibroblastic tumors, castleman disease, and Rosai-Dorfman disease, this patient was conclusively diagnosed with IgG4-RRD.

**Figure 3:**
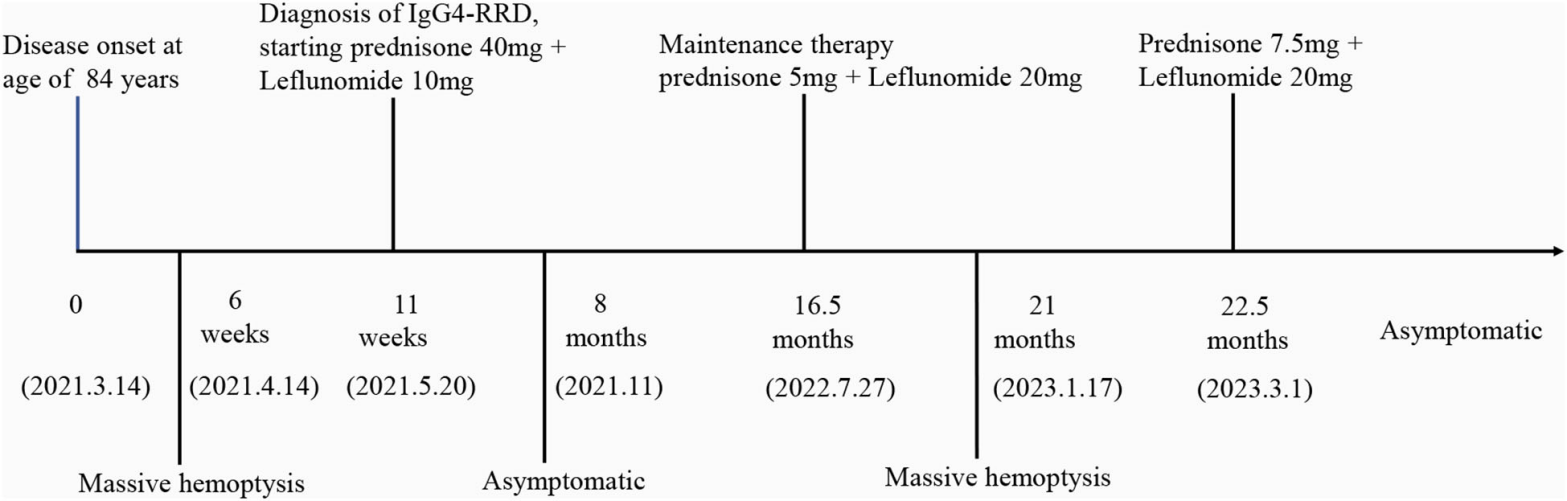
Timeline of the events. (IgG4-RRD, Immunoglobulin G 4 related respiratory disease).

Based on the recommendations from the Department of Rheumatology and Immunology, we prescribed oral prednisone 40 mg once daily, and oral leflunomide 10 mg once daily. The prednisone dose was tapered down slowly and maintained at 5 mg after 14 months, while leflunomide was gradually increased after 2.5 months and maintained at 20 mg once daily after 4.5 months. During the first six months of treatment, the patient occasionally had scant hemoptysis, which was managed with hemostatic medications and resolved with symptomatic treatment. Later, the symptoms almost completely disappeared, and serum IgG4 levels significantly decreased (**Figure 2B**). Follow-up chest CT scans at 16 months after treatment showed significant absorption of the lung lesions (**Figures 1C**). However, about 13 months after symptom resolution, the patient experienced intermittent hemoptysis again, including two episodes of massive hemoptysis, each amounting to approximately 100 ml. She was admitted to the hospital for the fourth time on January 17, 2023. A follow-up chest CT scan indicated a recurrence of the lesions (**Figure 1D**), and serum IgG4 levels was 990 mg/L, relatively stable compared to the previous assessment (**Figure 2B**). After treatment with vasopressin and phentolamine for hemostasis, the hemoptysis stopped. After excluding infections and neoplastic lesions, we increased the prednisone dose to 7.5 mg daily and continued leflunomide at 20 mg daily. The patient has not experienced any further hemoptysis since. A follow-up chest CT scan showed absorption of the lesions (**Figure 1D**). The timeline of events is presented in **Figure 3**.

## 3 Methods

We performed a literature review of published articles on IgG4-RD involving hemoptysis. We searched Pubmed using the following keywords: “Hemosputum”, “hemoptysis”, “bloody sputum”, “Phlegm Blood”, “Immunoglobulin G4”, “IgG4”, “Immunoglobulin G4 Related Disease”, “Immunoglobulin G4-Related Diseases”, “IgG4 Related Systemic Disease”, “IgG4-RD”, “IgG4-Associated Autoimmune Disease”, “Autoimmune Disease, IgG4-Associated”, “IgG4 Associated Autoimmune Disease”, “IgG4-Associated Autoimmune Diseases”, “IgG4-Related Disease”, “IgG4 Related Disease”, “IgG4-Related Diseases”. The literature was searched by two researchers. Finally, 12 articles (9–20) were included in the study (**Table 1**).

**Table 1:** Clinical characteristics of patients with hemoptysis involvement of IgG4-related diseases.

Authors	Type of study	N	Age/y	Serum IgG4	Quantity of hemoptysis	Other manifestations	Drug treatment	Outcomes
Sun X, et al. (9)	Retrospective study	2	18–71	53.8 % elevated, 46.2%normal	ND	—	Prednisone/Prednisone, Immunosuppressive agents	—
Saad MA, et al. (10)	Case report	1	13	Elevated	Massvie hemoptysis	Dyspnea, cough, night fever, decreased appetite, weight loss	Prednisolone, azathioprine, mycophenolate, rituximab	Died
Erlij D, et al. (11)	Case report	1	58	ND	ND	Hematuria, proteinura	Prednisolone	Recovered
Wu M, et al. (12)	Retrospective study	6	25-74	Elevated	ND	Cough, sputum, fever, chest tightness	Prednisolone/Prednisone, immunosuppressive agents	ND
Simon NL, et al. (13)	Case report	1	60	ND	ND	ND	oral corticosteroids	Recovered
Zheng YK, et al. (14)	Case report	1	79	Elevated	ND	hemoptysis, fever	Glucocorticoids, methotrexate, tripterygium wilfordii polyglycoside	Recovered
Jinnur PK, et al. (15)	Case report	1	60	Elevated	Blood-streaked sputum	Cough, whitish phlegm, shortness of breath	Prednisone, mycophenolate mofetil	Recovered
Kang MK, et al. (16)	Case report	1	63	Normal	ND	No	Oral glucocorticoids	Recovered
Tan H, et al. (17)	Case report	1	52	Elevated	Bloody sputum,10 mL fresh blood	Cough, fever, respiratory failure	Prednisolone	Recovered
Pifferi M, et al. (18)	Case report	1	15	Elevated	ND	No	Prednisolone	Recovered
Kitada M, et al. (19)	Case report	1	48	Elevated	Bloody sputum	No	ND	ND
Xie LJ, et al. (20)	Case report	1	65	Elevated	Bloody sputum	Cough	ND	ND
This case	Case reorpt	1	84	Elevated	Massvie hemoptysis	Cough, chest pain, shortness breath	Prednisolone, leflunomide	Recovered

N, number of patients; y, years; ND, not described.

## 4 Discussion

IgG4-RD is characterized by immune-mediated chronic inflammation and fibrosis. It is a rare condition with distinctive pathological features including extensive lymphoplasmacytic infiltration, storiform fibrosis, and obliterative phlebitis. The affected tissues often exhibit IgG4+ plasma cell infiltration and an elevated IgG4+/IgG+ plasma cell ratio. IgG4-RD is a systemic disease that can involve multiple organs and tissues either sequentially or simultaneously, such as the salivary glands, pancreas, lacrimal glands, orbital and periorbital tissues, lymph nodes, bile ducts, kidneys, thyroid, nervous system, retroperitoneum, mesentery, skin, liver, lungs, pleura, mediastinum, pericardium, arteries, breasts, and prostate (21, 22). The most frequently affected organs or anatomical sites include the pancreas, bile ducts, major salivary glands (submandibular and parotid), lacrimal glands, retroperitoneum, and lymph nodes. Clinical characteristics vary widely across patients with different organ involvement (23, 24), leading to frequent misdiagnosis and missed diagnosis.

IgG4-RRD is relatively uncommon compared to IgG4-RD involving other organs (3). Clinical symptoms are nonspecific, and most patients are asymptomatic. Their clinical manifestations vary depending on the site of involvement, with potential symptoms including cough, sputum production, dyspnea, chest pain, fever, asthenia, weight loss, and hemoptysis, which is relatively rare. A cohort study in China (5) found that only 1 out of 104 IgG4-RRD patients (1%) had hemoptysis, while 54.8% were asymptomatic, with cough being the most common symptom, accounting for approximately 25%. We conducted a literature review on IgG4-RD involving hemoptysis and found 12 published articles, describing 17 adult cases and 2 pediatric cases. Only one article (10) reported a 13-year-old child with IgG4-RD who had massive hemoptysis and ultimately died from it. Evidence suggests that IgG4-RD can affect the vascular system (25–28). Inflammatory aneurysms (29) and pulmonary arterial and venous endothelialitis (30) have previously been reported in IgG4-RD. Traction bronchiectasis caused by peripheral parenchymal fibrosis (31) and direct involvement of airway tissues (18) can both lead to hemoptysis. In this case, the patient’s chest CT did not reveal bronchiectasis, so we speculate that the massive hemoptysis may be due to plasma cell infiltration into the bronchial arteries.

IgG4-RD has diverse radiological features when affecting the chest. It can cause interstitial lung disease, lung infiltrates similar to bacterial pneumonia, lung nodules or masses, thickened bronchial walls, thickened bronchovascular bundles, and mediastinal/hilar lymphadenopathy (32). Pleural involvement is also common, presenting as chronic or recurrent pleural effusion with pleural nodules or diffuse thickening (32, 33). The effusion is usually exudative, with chylous effusion also reported (33–35). Inoue et al. (31) categorized the pulmonary imaging features of the disease into four types: solid nodular/mass, round ground-glass, alveolar interstitial, and bronchovascular bundle. These four types can occur alone or simultaneously in different combinations. The presence of a paravertebral soft band is relatively specific to the disease (36), corresponding to thickening of the paravertebral sulcus in the lower thoracic region, involving two or more vertebrae in succession. In this case, the pulmonary imaging primarily showed patchy density shadows, thickened bronchovascular bundles, paravertebral soft tissue shadows (**Figure 2C**), pleural effusion, and pleural thickening. Initially, a pulmonary tumor was suspected, but the final diagnosis of IgG4-RRD was confirmed by pathological biopsy. Notably, the size of the patchy shadows changed during multiple follow-up chest CT scans before the etiology-targeted treatment. We considered that this change might be related to concurrent bacterial infection since the inflammatory markers were elevated during the first two hospitalizations, and the condition improved with antibiotic treatment alone.

Serum IgG4 is an important indicator for diagnosing and assessing IgG4-RD. Most patients with IgG4-RD have elevated serum IgG4 levels, but some may present normal levels (37–39). Serum IgG4 concentration correlates with disease activity only in patients with elevated serum IgG4 concentrations (40). Almost all IgG4-RD patients with elevated serum IgG4 levels showed a significant reduction after treatment with steroids or other effective therapies, suggesting that immune inflammation is under control. This patient’s serum IgG4 was consistent with this pattern, with markedly elevated levels (3114 mg/L) at the initial diagnosis, which significantly decreased after treatment. However, during disease recurrence, the serum IgG4 remained stable and did not decrease further. Therefore, serum IgG4 levels should be monitored dynamically during maintenance treatment, and a lack of decrease or an increase warrants vigilance for disease recurrence.

Glucocorticoids are the first-line treatment for IgG4-RD, and combining immunosuppressant leflunomide with glucocorticoids is more effective than glucocorticoids alone in controlling the disease and reducing recurrence (41). There are no specific guidelines regarding dosage reduction and maintenance, and these must be determined based on individual patient conditions. This patient responded well to combined treatment with prednisone and leflunomide. However, during maintenance treatment with prednisone 5 mg and leflunomide 20 mg, the condition relapsed. Hemostatic medications effectively controlled the bleeding, and the disease was controlled again after adjusting the prednisone dose to 7.5 mg, with no recurrence since. Complications of IgG4-RD depend on the degree, location, and progression of fibrosis. This patient’s condition differs from the previously reported pediatric case (10), where a child died due to massive hemoptysis. The child initially improved on prednisolone, but azathioprine and then mycophenolate failed to control relapses during steroid tapering. Her last relapse was treated with rituximab but was ineffective, and she eventually died of massive hemoptysis. In this patient, massive hemoptysis occurred twice: once before treatment and once during maintenance therapy. Interestingly, both episodes of hemoptysis were effectively controlled with sufficient hemostatic medication. Particularly after recurrence, hemoptysis did not recur after adjusting the steroid dose, possibly because this patient was in the inflammatory stage, unlike the previous patient, who had likely progressed to the fibrotic stage. Unfortunately, when this 84-year old patient experienced the second episode of massive hemoptysis, no biopsy was performed to further monitor the changes in pathological progression. Therefore, early treatment is crucial to preventing irreversible organ damage caused by inflammation and fibrosis and is essential for patient prognosis.

## 5 Conclusions

We have provided a detailed report on the medical history, diagnosis, treatment, and consistent follow-up of an IgG4-RRD patient primarily presenting with massive hemoptysis. This is a rare form of IgG4-RD. To the best of our knowledge, this is the first report of an adult IgG4-RRD case involving massive hemoptysis. Clinical manifestations of IgG4-RRD are nonspecific, and pulmonary imaging findings are varied, leading to frequent misdiagnosis and missed diagnosis. When encountering patients with IgG4-RRD, particularly those with hemoptysis, the possibility of massive hemoptysis must be considered. Likewise, in patients with lung patch lesions and no evidence of a tumor on biopsy, IgG4-RRD should not be overlooked. Timely diagnosis and early treatment can improve patient prognosis.

## Data Availability

The original contributions presented in the study are included in the article/supplementary material. Further inquiries can be directed to the corresponding author.
